# Laryngeal suspension, combined with rehabilitation and nutritional support, improved the clinical course of a patient with sarcopenic dysphasia

**DOI:** 10.1016/j.ijscr.2020.04.068

**Published:** 2020-05-11

**Authors:** Ken Kasahara, Keisuke Okubo, Jun Morikawa

**Affiliations:** Department of Otorhinolaryngology, Sano Kosei General Hospital, 1728 Horigomecho, Sano City, Tochigi, Japan

**Keywords:** POD, postoperative day, Sarcopenic dysphasia, Malnutrition, Inactivity, Rehabilitation, Laryngeal suspension

## Abstract

**Introduction:**

Sarcopenic dysphasia is a relatively new disease concept describing impairments in swallowing resulting from a generalized loss of skeletal muscle mass.

**Presentation of case:**

In this case report, we describe the clinical history and presentation of a 76-year-old man who developed mild sarcopenic dysphasia following a period of physical inactivity after spinal stenosis surgery, which resulted in a loss of 10 kg of body weight in the 10-month period after surgery. The patient’s dysphasia was managed with laryngeal suspension, performed via a minimally invasive thyromandibulopexy, in combination with rehabilitation and nutritional support. After a brief period of postoperative rehabilitation, the patient was able to eat soft meals on postoperative day 14, and a regular meal on postoperative day 18, without aspiration. We include a brief description of our surgical technique in the case report.

**Discussion:**

Laryngeal suspension compensated for a decreased functional capacity of the swallowing muscles, with postoperative rehabilitation improving the strength of the swallowing muscles. Fixation of the thyroid cartilage to the mandible compensated for insufficient opening of the esophageal orifice, decreasing the pyriform sinus residue. Drawing of the thyroid cartilage in an anterosuperior position improved the anterosuperior position of the epiglottis, shortening the distance between the epiglottis and the base of the tongue, which narrowed the vallecula space and decreased vallecular residue.

**Conclusion:**

Based on our experience, laryngeal suspension, via minimally invasive thyromandibulopexy, could be considered to improve the outcomes of sarcopenic dysphagia, with an earlier return to eating normal meals.

## Highlights

• Sarcopenic dysphasia results from a generalized loss of skeletal muscle mass.
• We used laryngeal suspension to compensate for a decrease in swallowing function.
• Laryngeal suspension supported an earlier recovery to eating normal meals.
• Laryngeal suspension could improve outcomes of sarcopenic dysphagia.

## 1 Introduction

Sarcopenic dysphasia is a relatively new disease concept, describing impairments in swallowing resulting from a generalized loss of skeletal muscle mass [1,2]. The first position paper on sarcopenic dysphasia, published in January 2019, described the diagnostic criteria (age, hand grip strength, gait speed, general muscle mass, impaired swallowing, and absence of an obvious cause of dysphasia) and general therapeutic management, including resistance training of the swallowing muscles and nutritional support [3]. In this case report, we describe our use of laryngeal suspension, via a minimally invasive thyromandibulopexy, in combination with rehabilitation and nutritional support, for a patient with sarcopenic dysphasia, including the postoperative course of recovery of swallowing function.

This work has been reported in line with SCARE criteria(Agha) [4].

## 2 Case presentation

### 2.1 Clinical history

The patient was a 76-year-old man with a history of mild dysphasia. He had a prior history of cerebral infarctions (x3) at the age of 66, 74 and 75 years, with a first instance of swallowing difficulties after the third stroke. At that time, dysphasia rehabilitation was initiated within 3 days of the stroke, and he recovered the ability to eat a regular meal within 2 weeks. In January 2018, 1-year after his last stroke, he underwent surgery for spinal stenosis, followed by a 10-day period of bedrest. His physical activity level subsequently declined due to back pain, and he lost 10 kg within the 10 months after surgery. Swallowing difficulties re-emerged in September 2018, with the patient only able to tolerate soft meals. His dysphasia worsened, with an increase in sputum and development of a chronic cough.

### 2.2 Medical history and assessment of swallowing

**Fig. 1:**
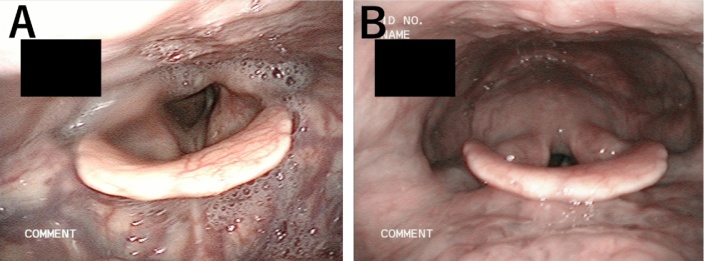
(A) Pre-operative video-endoscopic evaluation showing salivary penetration into the larynx and a wide pharyngeal space. (B) Postoperative video-endoscopic evaluation showing absence of saliva residue and a decrease in vallecula residue due to a shortened distance between the epiglottis and the base of the tongue, as well as a narrowing of the space of the vallecula.

**Fig. 2:**
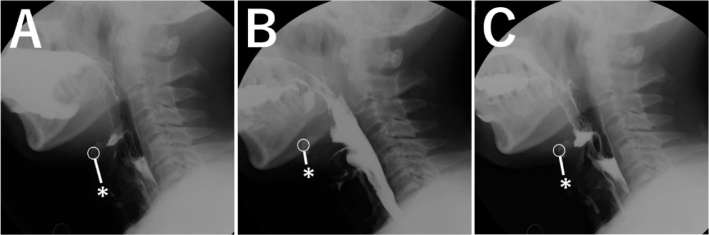
Pre-operative video-fluorography showing enlargement of the esophageal orifice, with a weak anterior movement of the larynx. The contraction of the pharyngeal constrictor muscle is decreased, with evident residue in the vallecula and pyriform sinus after swallowing and mixed aspiration. In (A)-(C), circles indicate the location of the hyoid bone. Asterisks indicate the location of the superior border of the thyroid cartilage.

His medical history included cerebral infarctions (x3), diabetes mellitus, spinal stenosis, and hypertension. His height was 168.8 cm, with a body weight of 56.5 kg. He had a right hemiplegia, with the following functional measures: grip strength, 25 kg; gait speed, 0.7 m/s, assessed using the 5-m walk test; and skeletal mass index, 6.6 kg/m^2^. His score of 5 points on the Mini Nutritional Assessment Short Form was indicative of malnourishment. Absence of another cerebral infarct was confirmed by magnetic resonance imaging. Video-endoscopic swallowing assessment showed saliva pooling in the pharynx, widening of the pharyngeal space, vallecular and pyriform sinus residue, and aspiration (Fig. 1A). Video-fluorography revealed a decrease in the forward movement of the larynx, a widened pharyngeal space, weakened pharyngeal constriction, vallecular and pyriform sinus residue, and aspiration (Fig. 2A–C). Functional swallowing scores were as follows: Hyodo score, 9; Fujishima grade, 6; FILS, 8; and dysphasia severity scale, 4. A probable diagnosis of sarcopenic dysphagia was made, based on the criteria defined by Mori et al. [5] Clinical management included swallowing training, a high-protein diet, and nutritional guidance. The patient declined additional rehabilitation, due to his previous experience post-stroke. Therefore, we presented laryngeal suspension as a possible alternative treatment, which was performed in December 2018.

### 2.3 Surgical procedure

**Fig. 3:**
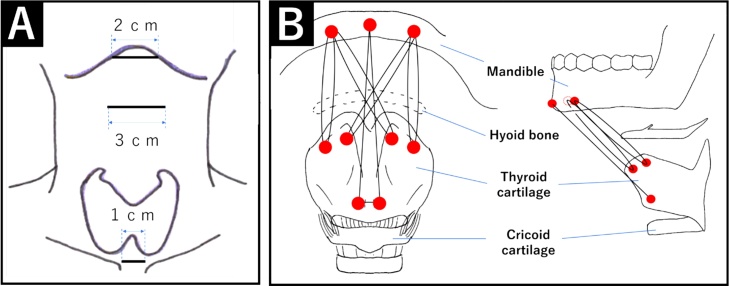
(A) Placement of the two skin incisions used for the minimally invasive laryngeal suspension, a 2-cm incision at the revel of the mandible and a second 3-cm incision between the hyoid bone and the superior border of the thyroid cartilage. A 1-cm skin incision is also performed for tracheostomy. (B) Three holes, each separated by 1 cm, are created in the mandible and 6, each separated by less than 1 cm, in the thyroid cartilage. The mandibular and the thyroid cartilage were closely fixed using No.2 nylon thread, passed through the subcutaneous tunnel on the hyoid bone.

Laryngeal suspension was performed via a minimally invasive thyromadibulopexy, using two skin incisions: one (2-cm) incision at the level of the mandible and the other (3-cm) between the hyoid bone and the superior border of the thyroid cartilage (Fig. 3A). Identified the white line, divided aside the anterior cervical muscles, and exposed the thyroid cartilage. Also the mandible was exposed. Two holes in the mandibular and six holes in the thyroid cartilage was made by using drill. The thyroid cartilage was fixed to the mandible using No. 2 nylon. No. 2 nylon thread was passed through the Gore-tex sheet cut to the size of thyroid cartilage. And it was passed through the thyroid cartilage, subcutaneous tunnel on the hyoid bone, mandible, subcutaneous tunnel on the hyoid bone, and tied to the other side of the nylon. As the two holes of the middle of the thyroid cartilage, two No. 2 nylons was crossed to fix the thyroid cartilage and mandible. The larynx was suspended about one vertebral body by totally using five No. 2 nylons (Fig. 3B), confirmed by X-ray fluoroscopy. A 1-cm incision was performed for tracheotomy, and cuffed tracheal cannula was inserted for airway management.

### 2.4 Postoperative course

**Fig. 4:**
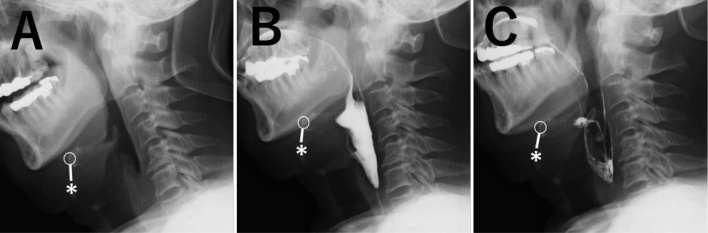
Postoperative video-fluorography showing the anterosuperior suspension of the larynx after surgery, with absence of pharyngeal residue of contrast agent and aspiration. In (A)-(C), circles indicate the location of the hyoid bone. Asterisks indicate the location of the superior border of the thyroid cartilage.

Maintaining some neck flexion for a period of 1-week was conducted. Nutritional support was initiated via gastrostomy tube feeding. The cuffed tracheal cannula was changed to an uncuffed cannula on postoperative day (POD) 1. The wound was stable and there was no pain. Swallowing was initiated using jelly and cold tea, with the patient in a flat lateral position. The tracheal cannula was removed on POD 4. Video-fluorography revealed contrast agent residue in the vallecular but with a decrease in residue in the pyriform sinus compared to pre-operative measures, with no evidence of aspiration on POD 6 (Fig. 4A–C). Anterosuperior movement of the larynx was improved, reaching one vertebral level higher than before surgery. The swallowing reflex was maintained, with sufficient opening of the esophageal orifice, avoiding the need for mandibular protrusion to swallow. Swallowing using modified food was initiated in a sitting position on POD 6 and the gastrostomy tube was removed on POD 7. Video-endoscopic examination confirmed his ability to eat a rice cracker and noodles without aspiration and no evidence of pharyngeal residue on POD 11 (Fig. 1B). The patient was able to eat soft meals on POD 14, and a regular meal on POD 18, and was discharged on POD 21. The patient gained 2 kg in the 2 months after surgery, with improvement in functional scores: Hyodo score, 5; Fujishima grade, 8; FILS, 8; and dysphasia severity scale, 6. There was no exacerbation of the condition over a 10-month period after surgery, and the patient has a normal diet and is able to travel. Neck movement restriction was not severe.

## 3 Discussion

Laryngeal suspension, combined with rehabilitation, shortened the recovery time of swallowing function in a patient with dysphagia resulting from age and physical activity limitation. The European Working Group on Sarcopenia in Older People describes primary sarcopenia resulting from aging, with secondary sarcopenia resulting from activity limitations, disease, and/or poor nutritional status [6,7], with the main cause of dysphasia attributed to physical inactivity, with a poor nutritional status and his age being contributing secondary factors [8]. There was no evidence of impairment in oral retention, bolus formation, transfer of the bolus to the pharynx, and delay in swallowing reflex which are typically seen with a neurological cause of dysphasia.

Malnutrition may be a contributing factor to dysphagia after a stroke [1]. Maeda and colleagues [9] reported on the development of sarcopenic dysphasia in the background of other diseases, which increases the difficulty of identifying the main cause of dysphasia. According to Wakabayashi et al. [10] determination of the presence or absence of an obvious cause is the most difficult step in the diagnosis of sarcopenic dysphasia, and sarcopenic dysphagia should be considered even in patients with a history of other possible causes of dysphagia.

Management of sarcopenic dysphagia requires resistance training of swallowing muscles and nutritional support [1,11,12]. Wakabayashi and colleagues [13] reported benefits of nutritional support (about 35 kcal/kg/day) and rehabilitation in improving bodyweight, muscle mass, strength, and dysphagia after right-upper lobe resection in a patient with lung cancer, with removal of the gastrostomy tube possible on POD 120. In our study, we demonstrate the possibility of using laryngeal suspension to shorten treatment time, which agrees with a previous report on laryngeal suspention [14]. While laryngeal suspension compensates for a decreased functional capacity of the swallowing muscles, postoperative rehabilitation improves the mass and strength of swallowing muscles, especially those associated with laryngeal suspension and pharyngeal constriction. In our patient, vallecula and pyriform sinus residue was caused by decreased strength of the suprahyoid muscles, reduced anterosuperior movement of the larynx, and insufficient opening of the esophageal orifice, as well as a decrease in the strength of the pharyngeal constrictor muscle, which lowers the swallowing pressure and leads to difficulties in opening of the esophageal orifice. Video-fluorography revealed further impairments in opening duration and timing of the cricopharyngeal muscle, but with no impairment in the relaxation phase of the muscle. Fixation of the thyroid cartilage to the mandible compensated for insufficient opening of the esophageal orifice, decreasing the pyriform sinus residue. Moreover, drawing of the thyroid cartilage in an anterosuperior position improved the anterosuperior position of the epiglottis, shortening the distance between the epiglottis and the base of the tongue, which narrowed the vallecula space and decreased vallecular residue.

As improving swallowing function in the short term is important to improve quality of life, of both the patient and caregivers, laryngeal suspension may be an effective adjunct to rehabilitation and nutritional support. This surgical approach could also decrease the length of hospital stay, with rehabilitation and nutritional support managed on an out-patient basis or in a community clinic. Minimally invasive thyromandibulopexy preserves the swallowing reflex, without requiring mandibular protrusion. We note that although cricopharyngeal myotomy was not included in our case, in the absence of impairment in opening of the esophageal orifice and relaxation of the cricopharyngeal muscle, it could be considered as a future step if there was evidence of increasing pharyngeal residue (due to a progressive decrease in muscle strength and swallowing pressure) and to prevent aspiration pneumonia.

However, although it is accepted that the severity of sarcopenic dysphasia will influence the length and outcome of rehabilitation, surgical indication and timing has not been clearly defined to date, with management of sarcopenic dysphagia largely considered on a patient-by-patient basis. In our case, the wound was stable and direct exercise, using modified food, was initiated on POD 4. Maintaining some neck flexion for a period of 1-week has been proposed to improve wound outcomes [15]; therefore, the timing of rehabilitation after surgery will also need consideration.

## 4 Conclusion

Based on our experience, laryngeal suspension, via minimally invasive thyromandibulopexy, could be considered to improve the outcomes of sarcopenic dysphagia, with an earlier return to eating normal meals. The effects of severity of dysphagia will need to be considered in the future.

## Declaration of Competing Interest

The authors have no conflicts of interest to declare.

## Sources of funding

No funding to declare.

## Ethical approval

This is a case report study; no ethical approval was needed.

## Consent

Written informed consent was obtained from the patient for publication of this case report and accompanying images. A copy of the written consent is available for review by the Editor-in-Chief of this journal on request".

## Author contribution

Keisuke Okubo: Second surgical assistant, postoperative management and proofreading of this paper.

Jun Morikawa: Third surgical assistant and postoperative management.

## Registration of research studies

1 Name of the registry: Research Registry
2 Unique identifying number or registration ID: researchregistry5533
3 Hyperlink to your specific registration (must be publicly accessible and will be checked): https://www.researchregistry.com/browse-the-registry#home/

## Guarantor

Ken Kasahara.

## Data availability statement

The data that support the findings of this study are available from the corresponding author, Kasahara, upon reasonable request.

## Provenance and peer review

Not commissioned, externally peer-reviewed.
